# Roles of cellular heterogeneity, intrinsic and extrinsic noise in variability of p53 oscillation

**DOI:** 10.1038/s41598-019-41904-9

**Published:** 2019-04-10

**Authors:** Dao-Guang Wang, Shaobing Wang, Bo Huang, Feng Liu

**Affiliations:** 1 National Laboratory of Solid State Microstructures, Department of Physics, and Collaborative Innovation Center of Advanced Microstructures, Nanjing University, Nanjing, 210093 China; 2 School of Physics and Electronic Engineering, Jiangsu Normal University, Xuzhou, 221116 China

## Abstract

The p53 protein is a key mediator of the cellular response to various stress signals. In response to DNA damage, the concentration of p53 can temporally oscillate with fluctuations in both the amplitude and period. The underlying mechanism for p53 variability is not fully understood. Here, we construct a core regulatory network of p53 dynamics comprising the ATM-p53-Wip1 and p53-Mdm2 negative feedback loops. We dissect the contributions of cellular heterogeneity, intrinsic noise, and multiple forms of extrinsic noise to p53 variability in terms of the coefficients of variation of four quantities. Cellular heterogeneity greatly determines the fraction of oscillating cells among a population of isogenic cells. Intrinsic noise—fluctuation in biochemical reactions–has little impact on p53 variability given large amounts of molecules, whereas extrinsic colored noise with proper strength and correlation time contributes much to oscillatory variability in individual cells. With the three sources of noise combined, our results reproduce the experimental observations, suggesting that the long correlation time of colored noise is essential to p53 variability. Compared with previous studies, the current work reveals both the individual and integrated effects of distinct noise sources on p53 variability. This study provides a framework for exploring the variability in oscillations in cellular signaling pathways.

## Introduction

The p53 protein is one of the most important tumor suppressors, mediating a multitude of cellular responses to stress including DNA damage and oncogene activation^1,2^. The subcellular localization, posttranslational modifications, concentration, and dynamics of p53 all affect its tumor suppressive function. It is increasingly evident that p53 dynamics play a key role in cell fate decision^3,4^. Upon DNA damage, the concentration of p53 exhibits oscillatory behavior in various cell lines such as MCF-7, H1299 and U2OS ones^4–8^, and it was suggested that the number of p53 pulses determines cellular outcome^9,10^. Notably, there exists variability in both the amplitude and period of p53 oscillations at the single-cell and population levels^11^. It was proposed that such variability originates from intrinsic and extrinsic noise in the p53 signaling network^11,12^ and from cellular heterogeneity^13^.

Although the impacts of intrinsic noise^13–15^ and extrinsic noise^11^ on the variability in p53 oscillation have been probed respectively, an integrative study is missing; combining various sources of noise and comparing the relative influence of each source should unravel the essence of p53 variability and its influence on the function of p53. This in turn provides an effective manner to modulate p53 dynamics and enhance the design of p53-based therapeutic treatments.

Motivated by the above considerations, here we build a minimal network model, comprising the essential regulators of p53 dynamics, and quantify the effect of noise on p53 variability in terms of the coefficients of variation of the amplitude, width, period of p53 oscillations and the delay between p53 and Mdm2 oscillations. We first examine the deterministic dynamics of the network and the influence of cellular heterogeneity on oscillatory dynamics, then probe the stochastic dynamics of the network and the effects of intrinsic and extrinsic noise on oscillatory variability, and further integrate the three sources of noise and reveal their combinatory impact on p53 variability. We use multiple methods to estimate the noise parameters, which is important for the interpretation of experimental data. In contrast to a minor effect of intrinsic noise on p53 variability given large numbers of species, cellular heterogeneity greatly determines the fraction of oscillating cells, and the extrinsic noise with long correlation time underlies the p53 variability in individual cells. Since the stochastic factors we analyze are ubiquitous in biochemical systems, our main conclusions may be of wide applicability, and our modeling approach could be exploited to investigate the stochasticity in biochemical systems.

## Model and method

### Model

**Figure 1:**
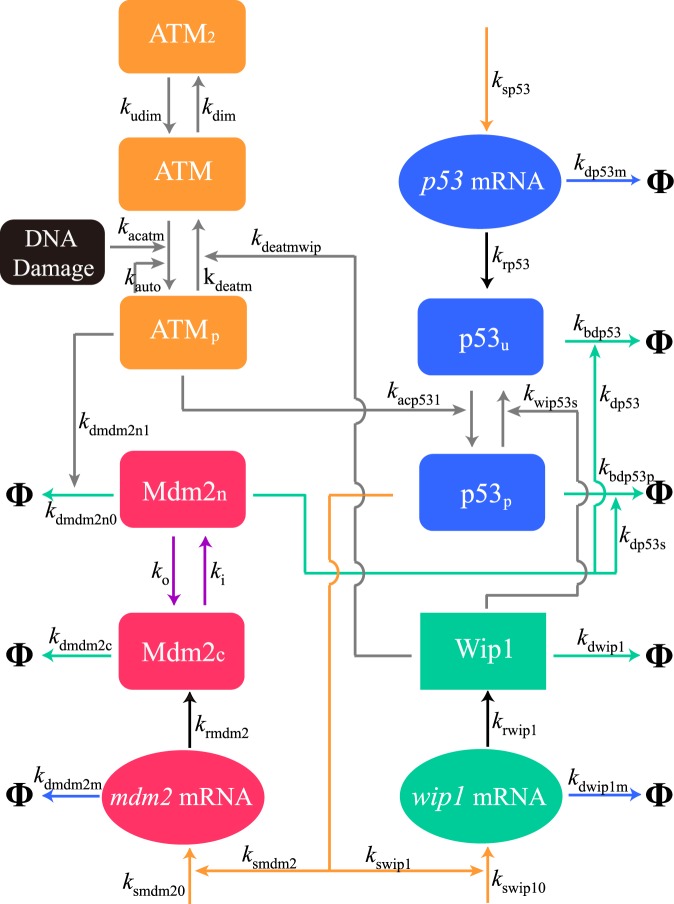
Diagram of the network model. Upon DNA damage, ATM is activated via autophosphorylation, and ATM_p_ further phosphorylates p53 and Mdm2, stabilizing and activating p53. p53_p_ induces the expression of *mdm2* and *wip1*, while Wip1 dephosphorylates ATM_p_ and p53_p_. Lines with arrow head denote the processes of transcription (orange), translation (black), mRNA degradation (blue), protein degradation (green), reversible phosphorylation (gray), and nuclear import and export (purple). Φ denotes the degradation of species. All the reaction rate constants are marked. p53_u_: unphosphorylated p53; p53_p_: phosphorylated p53; Mdm2_n_: nuclear Mdm2; Mdm2_c_: cytoplasmic Mdm2.

Based on previous studies^8–11^, we construct a minimal network model, comprising the core components regulating p53 oscillation (Fig. 1). Under unstressed conditions, p53 is kept at low levels due to the negative regulation by the E3 ubiquitin ligase Mdm2^16,17^. When cells are exposed to ionizing radiation (IR) or DNA-damaging agents such as neocarzinostatin (NCS), DNA double-strand breaks (DSBs) are induced. Normally, DNA repair proteins are recruited to fix the DSBs, thus leading to a limiting number of p53 pulses^6^. Several theoretical models have taken into account the DNA repair process, aiming to unravel the dependence of cell fate on the extent of DNA damage^9,10^. Since we only focus on the variability in oscillatory dynamics, especially in the amplitude and period, here we ignore the DNA repair process and take the number of initial DSBs as an input. Such a setting allows us to simulate long-term dynamics and sample sufficient pulses for statistical analysis.

The ataxia mutant (ATM) kinase is activated via rapid autophosphorylation upon IR, with ATM dimer (ATM_2_) converted to phosphorylated ATM monomer (ATM_p_)^18–20^. ATM_p_ phosphorylates p53 and Mdm2, promoting the degradation of Mdm2 and enhancing the stability and activity of p53^21–23^. Phosphorylated p53 (p53_p_) induces the production of Mdm2 and the phosphatase Wip1 (PPM1D), and Wip1 dephosphorylates p53_p_ and ATM_p_^24,25^. Thus, there exist three negative feedback loops: the p53-Mdm2, p53-Wip1 and ATM-p53-Wip1 loops. Although it was reported that, as a target of p53, PTEN can weaken the Mdm2-mediated inhibition of p53 by repressing Akt activity^26^, PTEN cannot always be expressed, such as in MCF7 cells because of its promoter methylation^27^. Given our model is a minimal one, here we ignore PTEN and the associated positive feedback loop. Moreover, mRNAs are explicitly included here.

### Deterministic equations

In the continuum limit, the dynamics of the network are governed by ordinary differential equations (ODEs). We mathematicalize our model in a manner similar to that in refs ^9,10^. (see Supplementary Information (SI) Text for details). The reaction rate constants in the ODEs are marked in Fig. 1. All the default parameter values and initial values of variables are listed in Tables S1 and S2, respectively. Time is in units of minutes, while the concentrations of network components are dimensionless, i.e., the number of species is divided by dimensionless cell volume, Ω (see *SI Text* for details). Moreover, we take into account cellular heterogeneity and set different values for reaction rate constants among a population of isogenic cells (see below).

### Stochastic simulation

Generally, the stochasticity in biochemical processes can be described by intrinsic and extrinsic noise. The intrinsic noise–fluctuations in biochemical reactions–is significant when the number of reactant molecules is small (e.g. less than 10^2^). Extrinsic noise has many sources. One source is the time-varying fluctuation in the amounts of species that do not appear explicitly in the model but still participate in reactions, e.g., RNA polymerases controlling the rate of transcription and ribosomes regulating the rate of translation^28^. Extrinsic noise is often colored, i.e., temporally correlated. In contrast to uncorrelated noise (white noise) with zero correlation time, correlated noise (colored noise) has a finite (non-zero) correlation time^29–31^. To unravel the impact of intrinsic and extrinsic noise as well as their combination on network dynamics, we perform three types of stochastic simulation.

1. For the simulation of intrinsic noise alone, we transform the above ODEs into a set of coupled biochemical reactions given in Table S3. The reaction rates are also listed there. Stochastic simulations are performed using the binomial *τ*-leap algorithm^32^, rather than the Gillespie one^33,34^. The reason is as follows. For a large number of reactants, the *τ*-leap algorithm is more efficient since reactions may occur more than once every time step^35^. More exactly, the number of times that each reaction occurs obeys a Poisson distribution with the mean equaling the firing rate times the time step. For some species, however, its number could be negative if the possible decrease in molecule number due to reactions like degradation exceeds the increase plus the current number. This problem is solved by using the binomial *τ*-leap algorithm, which replaces the Poisson distribution with a binomial distribution (see *SI Text* for details). The time step is fixed at Δ*t* = 0.01 min.
2. For the simulation of extrinsic noise in a specific process (e.g., gene transcription, translation, degradation of protein or mRNA), we assume that relevant parameters (see Table S4) change stochastically. Take the transcription of *p53*, *mdm2* and *wip1* as an example. In simulation, we solve the ODEs using Euler method with time step Δ*t* = 0.01 min; at each step, *k*_sp53_ is multiplied by ${e}^{{\eta }_{1}(t)}$, *k*_smdm20_ and *k*_smdm2_ are multiplied by ${e}^{{\eta }_{2}(t)}$, and *k*_swip10_ and *k*_swip1_ are multiplied by ${e}^{{\eta }_{3}(t)}$, where *η*_*i*_(*t*) (*i* = 1, 2, 3) are assumed to be independent uniform white noise (UWN), Gaussian white noise (GWN), or colored noise (CN). For the UWN, $\eta (t)\sim {\mathscr{U}}(\,-\,a,a)$ (*a* > 0), where ${\mathscr{U}}(\,-\,a,a)$ denotes the uniform distribution with the probability density being $\frac{1}{2a}$ over [−*a*, *a*] or zero otherwise; for the GWN, $\eta (t)\sim {\mathscr{N}}\mathrm{(0},{\sigma }^{2})$, where ${\mathscr{N}}\mathrm{(0},{\sigma }^{2})$ denotes the normal distribution with mean 0 and variance *σ*^2^. The samples of *η*(*t*) at each step are independent, because white noise is uncorrelated temporally.
  Colored noise can be generated by the Ornstein-Uhlenbeck (OU) process, obeying the following stochastic differential equation:
  1 $$\frac{d\eta (t)}{dt}=-\frac{\eta (t)}{\tau }+{c}^{\mathrm{1/2}}{\rm{\Gamma }}(t),$$
  where Γ(*t*) is Gaussian white noise, *τ* is the relaxation time, and *c* is the diffusion constant^36,37^. We assume $c=\frac{2D}{\tau }$ with *D* denoting the strength of noise. Thus, the OU process with the initial condition *η*(*t*_0_) = *η*_0_ is^38^
  2 $$\eta (t)={\mathscr{N}}({\eta }_{0}{e}^{-(t-{t}_{0})/\tau },D(1-{e}^{-2(t-{t}_{0})/\tau }))(t\ge {t}_{0}),$$
  where ${\mathscr{N}}(m,{\sigma }^{2})$ denotes the normal random variable with mean *m* and variance *σ*^2^. Specifically, for a stationary OU process, the distribution of *η*(*t*) is time-independent, and $\eta (t)={\mathscr{N}}(0,D)$. That is, *D* is just the variance of the stationary distribution of *η*(*t*). The autocovariance function is
  3 $${\rm{cov}}\{\eta (t),\eta (t^{\prime} )\}=D{e}^{-(t^{\prime} -t)/t},$$
  with *t*′ > *t*. Thus, *η*(*t*) and *η*(*t*′) are significantly correlated only when *t*′ − *t* is much less than *τ*, which is thus called correlation time. Based on Eq. (2), *η* is updated at each time step^38,39^:
  4 $$\eta (t+{\rm{\Delta }}t)={e}^{-{\rm{\Delta }}t/\tau }\eta (t)+{[D(1-{e}^{-2{\rm{\Delta }}t/\tau })]}^{1/2}\xi ,$$
  where Δ*t* = 0.01 min is the time step and *ξ* is a unit normal random variable.
3. For the simulation of both the intrinsic and extrinsic noise, we integrate the two methods above. At each step in the binomial *τ*-leap algorithm, some parameters are multiplied by *e*^*η*(*t*)^. Specifically, the colored noise *η* is generated based on Eq. (4) with Δ*t* = 0.01 min.

## Results

### p53 oscillation in the deterministic case

**Figure 2:**
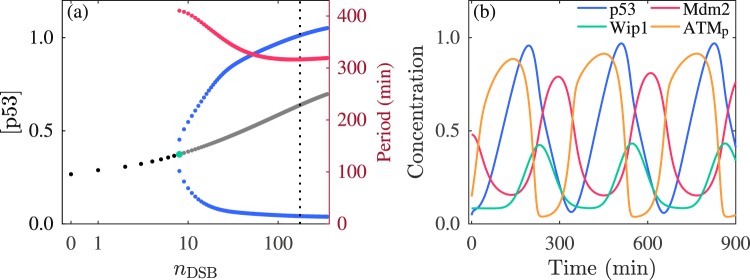
Network dynamics without noise. (**a**) Bifurcation diagram of [p53] versus *n*_DSB_. Black and gray dots denote the stable and unstable steady states, respectively. The blue dots denote the maxima and minima of p53 oscillation. The vertical dotted line indicates *n*_DSB_ = 175. The red dots denote the oscillation period (right *y*-axis). (**b**) Time courses of protein concentrations at *n*_DSB_ = 175. [p53] and [Mdm2] denote the total concentration of unphosphorylated and phosphorylated p53 and that of nuclear and cytoplasmic Mdm2, respectively.

We first investigate the dynamics of the network in the absence of any noise. Without DSB, i.e., *n*_DSB_ = 0, the network remains in a stable steady state with [p53] kept at low levels (Fig. 2(a)). At *n*_DSB_ = 8, the steady state becomes unstable and limit-cycle oscillation arises. As *n*_DSB_ increases, the amplitude and period of p53 oscillation first rise and drop, respectively, and then change slightly. It is known that 35 DSBs on average can be induced upon the irradiation of 1 Gy^40^. Since our results will be compared with the experimental data in ref. ^11^, where the irradiation of 5 Gy is applied, we set 175 as the default value of *n*_DSB_ in the following analysis. At *n*_DSB_ = 175, the oscillation period is 317 min (~5.3 h), consistent with the experimental results^8,41^.

Notably, ATM is first activated by DSBs (Fig. 2(b)), leading to a rise in [ATM_p_], and ATM_p_ phosphorylates p53 and Mdm2. Phosphorylated Mdm2 is then degraded rapidly. Consequently, p53 is stabilized and activated to induce the production of Wip1 and Mdm2; Wip1 in turn dephosphorylates ATM_p_ and p53_p_, while Mdm2 targets p53 for degradation. These lead to a decrease in [ATM_p_] and [p53]. ATM can be reactivated by DSBs, and a new round of oscillation is induced. Collectively, the ATM-p53-Wip1 and p53-Mdm2 negative feedback loops underlie the sustained oscillation of p53.

### Cellular heterogeneity

**Figure 3:**
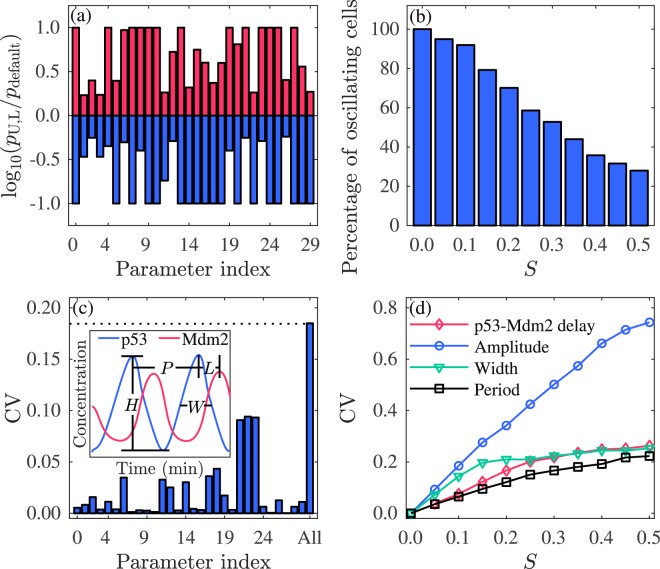
Influence of cellular heterogeneity on p53 oscillation. (**a**) Base-10 logarithm of the ratio of the upper (red) or lower (blue) threshold of each parameter admitting p53 oscillation to its default value. The parameter value ranges from 0.1 to 10 times its default value. Parameters are indexed as follows: 0. *n*_DSB_, 1. *k*_dwip1_, 2. *k*_rwip1_, 3. *k*_dwip1m_, 4. *k*_swip1_, 5. *k*_swip10_, 6. *k*_dmdm2n1_, 7. *k*_dmdm2n0_, 8. *k*_i_, 9. *k*_o_, 10. *k*_dmdm2c_, 11. *k*_rmdm2_, 12. *k*_dmdm2m_, 13. *k*_smdm2_, 14. *k*_smdm20_, 15. *k*_dp53s_, 16. *k*_bdp53p_, 17. *k*_dp53_, 18. *k*_bdp53_, 19. *k*_acp531_, 20. *k*_wip53s_, 21. *k*_rp53_, 22. *k*_dp53m_, 23. *k*_sp53_, 24. *k*_udim_, 25. *k*_dim_, 26. *k*_deatmwip_, 27. *k*_deatm_, 28. *k*_auto_, and 29. *k*_acatm_. (**b**) Percentage of oscillating cells versus *S*. (**c**) CV for the amplitude of p53 oscillation when each parameter alone (0–29) or all parameters (All) are perturbed at *S* = 0.1. The dotted line denotes the estimate of the CV in the latter case. The inset schematically shows the amplitude (*H*), width at half height (*W*), period (*P*) of p53 pulses and the p53-Mdm2 delay (*L*). (**d**) CVs for the amplitude, width, period of p53 oscillation and p53-Mdm2 delay versus *S*. All parameters are perturbed.

Given the heterogeneity among a population of isogenic cells, such as cell-to-cell variability in *n*_DSB_ and rates of protein synthesis and degradation^13^, we determine the upper and lower thresholds (*p*_U_ and *p*_L_) of 30 parameters, including *n*_DSB_ and 29 reaction rate constants, allowing for p53 oscillation when they separately vary from 0.1 to 10 times their default value *p*_default_ (see Table S1). We show the ratio of *p*_U_ (*p*_L_) to *p*_default_ on a logarithmic scale in Fig. 3(a). The system can still undergo oscillation even if any of those parameters is decreased by 40% or increased by 70%. That is, p53 oscillation can exist in a relatively wide parameter range.

To simulate cellular heterogeneity, we perturb *n*_DSB_ and each rate constant (denoted by *p*_*i*_ (*i* = 0, 1, 2, ..., 29)) via multiplying it by ${e}^{S{\xi }_{i}}$, where ${\xi }_{i}^{\prime} {\rm{s}}$ are independent unit normal random numbers, i.e., ${\xi }_{i}\sim {\mathscr{N}}(0,1)$, and *S* describes the degree of parameter heterogeneity^12,13^. For each *S*, we randomly sample 1000 parameter sets (all ${p}_{i}^{\prime} {\rm{s}}$ are perturbed simultaneously) and calculate the percentage of parameter sets admitting p53 oscillations (Fig. 3(b)). The percentage drops with increasing *S*: p53 oscillations always occur at *S* = 0, but appear for only 33.6% of parameter sets at *S* = 0.5.

To characterize the influence of cellular heterogeneity on p53 oscillation, we calculate the coefficients of variation (CVs) of the amplitude, width, and period of p53 oscillation and the delay between p53 and Mdm2 oscillations (as depicted in the inset of Fig. 3(c)). The CV is defined as the ratio of standard deviation to mean of statistical variables. When only one of 30 parameters is perturbed and the others are kept at their default values, all the CVs at *S* = 0.1 are lower than 0.1, and relatively high CVs are induced when *k*_sp53_, *k*_dp53m_, or *k*_rp53_ is perturbed (Figs. 3(c) and S1(a)–(c)). These three parameters modulate the production and degradation of *p53* mRNA and production of p53, respectively. Such sensitivity is consistent with the essential role of p53 in regulating network dynamics.

With all the parameters perturbed simultaneously, each CV is close to the root of sum of squares of the corresponding CV_*i*_ calculated when only one parameter is perturbed alone. Take the CV of the amplitude as an example. When only parameter *p*_*i*_ is perturbed, the oscillation amplitude is *μ*_*i*_ + *δ*_*i*_, where *μ*_*i*_ is the mean amplitude and *δ*_*i*_ is a random variable with zero mean and standard deviation *σ*_*i*_. The corresponding CV is thus C*V*_*i*_ = *σ*_*i*_/*μ*_*i*_. When all the parameters are perturbed simultaneously, the amplitude is *μ* + *δ*, where *μ* is the mean amplitude and *δ* is a random variable with zero mean and standard deviation *σ*. Ideally, all the mean amplitudes are identical (possibly equal to the amplitude with the default parameters), i.e., *μ*_*i*_ = *μ* for all *i*, and $\delta ={\sum }_{i}{\delta }_{i}$; for independent random variables, ${\sigma }^{2}={\sum }_{i}{\sigma }_{i}^{2}$. Thus, ${{\rm{CV}}}_{{\rm{All}}}^{2}={\sum }_{i}{{\rm{CV}}}_{i}^{2}$. Actually, this equality only approximately holds true, since *μ*_*i*_ may deviate from *μ* and ${\delta }_{i}^{\prime} {\rm{s}}$ are not completely independent.

When all the parameters are perturbed simultaneously, all the CVs rise with increasing *S* for *S* ≤ 0.5 (Fig. 3(d)). The CV of the amplitude is always the highest and differs from the other CVs more markedly at larger *S*. This indicates that the amplitude is more sensitive to parameter variation than the period, in agreement with experimental observation^11^.

### Intrinsic noise

**Figure 4:**
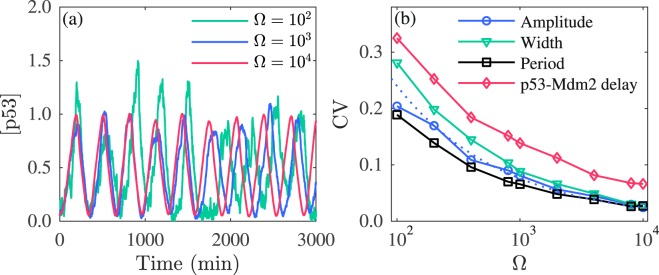
Impact of intrinsic noise on p53 oscillation. (**a**) Typical time courses of [p53] for Ω = 10^2^,10^3^ and 10^4^. (**b**) CVs of the amplitude, width, period of p53 oscillation and p53-Mdm2 delay versus Ω. The dotted curve is a fit to the data on the blue curve by 2.4 Ω^−0.5^. For each Ω, 1000 runs of simulations lasting 3000 min are performed. The axis of Ω is on logarithmic scale.

To probe the influence of intrinsic noise on the oscillatory dynamics, stochastic simulation is performed to obtain the temporal evolution of molecule numbers. The concentration of species equals its molecule number divided by Ω, which controls the system size and intensity of intrinsic noise. When Ω rises from 10^2^ to 10^4^, the fluctuations in p53 oscillations decrease increasingly (Fig. 4(a)). Accordingly, the CVs all drop monotonically with increasing Ω; for example, the CV of the amplitude declines approximately following 2.4 Ω^−0.5^ (Fig. 4(b)). A similar relationship was observed experimentally in a synthetic genetic oscillator^31^.

### Extrinsic colored noise

**Figure 5:**
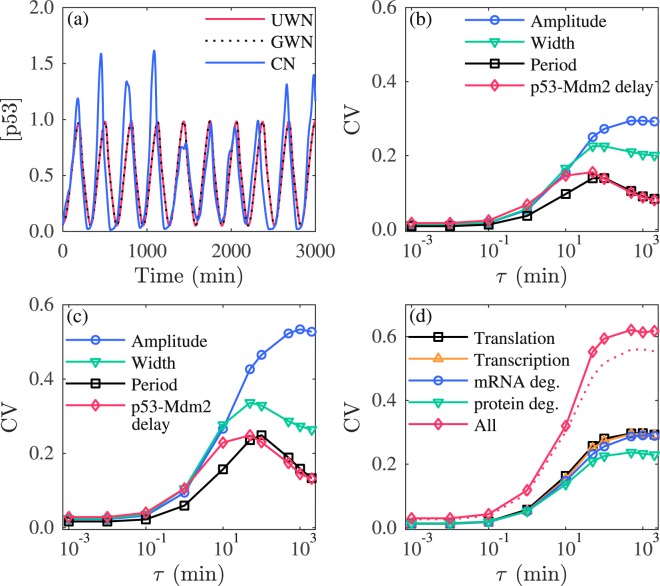
Influence of extrinsic noise on the variability in oscillations. (**a**) Time courses of [p53] with the UWN, GWN or CN at *D* = 0.1 and *τ* = 45 min. (**b**,**c**) Dependence of the CVs of the amplitude, width, and period of p53 pulses and p53-Mdm2 delay on *τ* at *D* = 0.1 (**b**) and *D* = 0.3 (**c**). (**d**) Comparison of the CV of the amplitude when the CN is added to the process of transcription, translation, mRNA degradation, protein degradation alone or together at *D* = 0.1. The dotted line denotes the estimate for the CV. In each case, 1000 runs of stochastic simulations lasting 3000 min are performed. The axis of *τ* is on logarithmic scale.

We model the effects of three types of extrinsic noise–UWN, GWN and CN–on gene transcription by perturbing the transcription rate constants in a similar manner to that in ref.^29^. In each case, the parameters relevant to the transcription of *p53*, *mdm2* and *wip1* (parameters on the orange lines with arrow in Fig. 1) are perturbed by independent noise (see Table S4). For comparison, three types of noise are assumed to have the same variance, i.e., *D* = 0.1 for CN, $a=-\sqrt{3D}$ for UWN, and *σ*^2^ = *D* for GWN. Notably, adding the UWN or GWN with small *D* does not markedly elevate oscillatory variability, whereas adding the CN with *τ* = 45 min leads to remarkable fluctuations in p53 oscillation (Fig. 5(a)). In fact, the UWN/GWN with *D* ≤ 1 has a minor impact on p53 variability (Fig. S2); but when *D* rises to around 2, the CVs begin to rise sharply, especially for the CV of the period. Furthermore, p53 oscillation even disappears for *D* > 2.2. We take the GWN as an example to interpret these results. At each time step the specific rate constants are perturbed by multiplying their default values with ${e}^{{\eta }_{i}(t)}$ (*i* = 1, 2, 3), where ${\eta }_{i}^{\prime} {\rm{s}}$ are independent and identically distributed Gaussian random variables with mean 0 and variance *D*. The expectation of ${e}^{{\eta }_{i}(t)}$ is *e*^*D*/2^, which equals 1.16 and 2.71 for *D* = 0.3 and 2, respectively. Thus, the mean values of perturbed parameters change slightly with *D* = 0.3 but vary a lot with *D* = 2, approaching the thresholds for oscillation. As a result, the system becomes more sensitive to noise intensity. Given the CN exerts a much larger effect on p53 variability compared to the UWN/GWN with the same variance, we only consider the CN with small *D* in the following.

We calculate the CVs for different values of *D* when *τ* is fixed at 100 min, where nearly the maximal CVs are acquired for each *D*. In the presence of only extrinsic noise, the noise with *D* around 0.2 induces a moderate CV. For *D* > 0.5, p53 oscillation will become rather irregular and it cannot be distinguished from purely random fluctuations. Thus, here we only present the results *for D* = 0.1 and *D* = 0.3 as examples. For *D* = 0.1, the CVs of the amplitude, width and period of p53 oscillation and p53-Mdm2 delay all change non-monotonically with increasing *τ* (Fig. 5(b)). If *D* is increased to 0.3, all the CVs become greater and still vary non-monotonically with *τ* (Fig. 5(c)). When *τ* is very small (e.g., *τ* = 0.001 min), the CN approximates the GWN with the same variance *D*, exerting little influence on oscillatory variability. Clearly, with such noise the fluctuations in reaction rate constants at adjacent time points tend to offset each other, leading to minor variability. In contrast, for the noise with relatively large *τ*, the changes are closely correlated within the time bin of *τ* and may differ markedly between adjacent bins, which promotes the variability in oscillation. When *τ* is sufficiently large, however, the long-range correlation instead weakens the variability, since the rate constants may change slightly over a long time interval. Collectively, the correlation time of the CN is crucial to the variability in p53 oscillation.

Similar to the CN in transcription, the CN in translation, mRNA degradation or protein degradation (by perturbing the parameters on the black, blue, and green lines with arrow in Fig. 1; see Table S4) all induces a similar non-monotonic change in CVs with increasing *τ* (Fig. 5(d) and Fig. S3(a)–(c)). If the CN is added to all the above four processes simultaneously (by perturbing the above parameters with 12 independent noise; see Table S4), the resulting CV of the amplitude still varies non-monotonically with *τ* and approximately equals the root of sum of squares of the CV’s above when *τ* is not large enough.

### Combinatory effect of intrinsic noise, extrinsic noise and cellular heterogeneity on p53 oscillation

**Figure 6:**
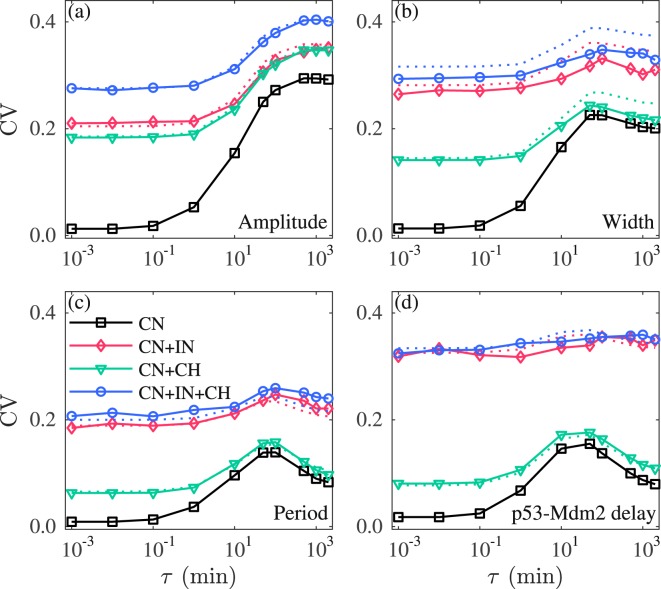
Combinatory effects of intrinsic noise (IN), extrinsic colored noise (CN) and cellular heterogeneity (CH) on the variability in oscillation. The influence of the CN alone, CN and IN, CN and CH, and three sources together on the amplitude (**a**), width (**b**), period (**c**) of p53 pulses and p53-Mdm2 delay (**d**). The dotted lines denote the estimates of CV. Parameters are set as follows: *D* = 0.1 for CN, Ω = 10^2^ for IN, and *S* = 0.1 for CH. In each case, 1000 runs of stochastic simulations lasting 3000 min are performed. In the presence of CH, a set of parameter values is sampled for each simulation. The CN is only added to the process of gene transcription. The axis of *τ* is on logarithmic scale.

We have shown how cellular heterogeneity, intrinsic and extrinsic noise each affect the variability in p53 and Mdm2 oscillations. Here, we explore their combinatory effect in three cases: the CN together with intrinsic noise (IN), CN with cellular heterogeneity (CH), and all three types of noise present, with *D* = 0.1, Ω = 10^2^, and *S* = 0.1. In each case, the CVs still vary non-monotonically with increasing *τ*, although they tend to be flat when more types of noise are added (Fig. 6). Notably, the CVs in the case of three types of noise combined can be well approximated by the root of sum of squares of the corresponding CV’s under individual noise sources.

Of note, the values of CVs in Fig. 6, especially that for the amplitude, differ from the experimental data in ref.^11^. To eliminate this discrepancy, we need to choose appropriate values of *S*, Ω, *D* and *τ* based on the known data. It was reported experimentally that nearly 50% of cells exhibit p53 oscillations upon irradiation of 5 Gy, and the CVs are 0.7 ± 0.05, 0.3 ± 0.05, 0.2 ± 0.05, and 0.3 ± 0.05 (mean ± standard deviation) for the amplitude, width, period and p53-Mdm2 delay, respectively^11^. According to our analysis of cellular heterogeneity, 52.8% of parameter sets allow for sustained oscillation at *S* = 0.3 (Fig. 3(b)). Accordingly, the CVs are 0.50, 0.22, 0.17, and 0.22 for the amplitude, width, period, and p53-Mdm2 delay; all these values are lower than the experimental values except the CV of the period. Moreover, cellular heterogeneity alone cannot underlie the irregularity in oscillation in individual cells. Therefore, part of p53 variability should be attributed to dynamic noise.

It was estimated that the number of p53 proteins in MCF7 cells is about 10^4^–10^5 ^^42^ and those of Mdm2 proteins and mRNA are around 10^5^ and 10^3^, respectively^43^. Given Ω = 10^4^, the numbers of proteins and mRNAs are around 10^3^–10^4^ and 10^2^–10^3^, respectively. Strikingly, in the presence of only intrinsic noise with Ω = 10^4^, the CVs are 0.024, 0.029, 0.027 and 0.066 for the amplitude, width, period, and p53-Mdm2 delay, which are all less than 0.1. That is, intrinsic noise alone exerts little effect on the oscillatory variability. Thus, extrinsic noise should play a marked role in p53 variability.

**Figure 7:**
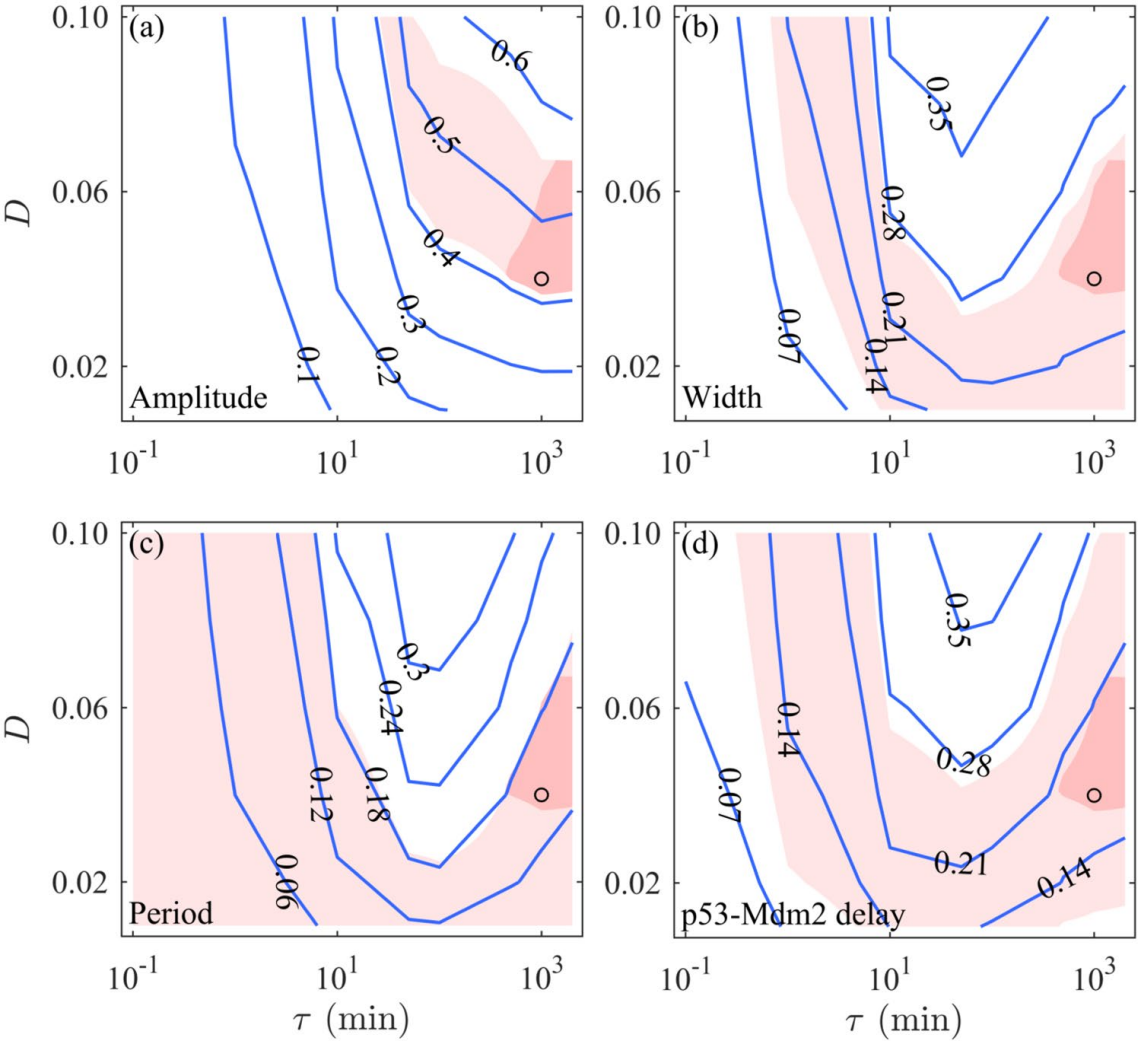
Estimation of the values of *D* and *τ*. Contour maps of *D* versus *τ* for the amplitude (**a**), width (**b**), period (**c**) and p53-Mdm2 delay (**d**) in the presence of only colored noise. Blue curves are specified by the values of CVs. The black circles denote the case with *D* = 0.04 and *τ* = 10^3^ min. In each case, 1000 runs of stochastic simulations lasting 3000 min are performed. The axis of *τ* is on logarithmic scale.

It is difficult to determine the values of *D* and *τ* directly due to lack of experimental data. But we make an estimation using the above approximate relationship between the resulting CV and CV’s under individual noise sources. Given *S* = 0.3 and Ω = 10^4^, the CVs under extrinsic noise alone should fall within the following ranges: [0.41,0.56], [0.11,0.27], [0,0.18] and [0.10,0.27] for the amplitude, width, period, and p53-Mdm2 delay, respectively. By parameter sweeps, we can roughly determine the ranges of *D* and *τ*. We run simulations for different *D* and *τ* when colored noise is added to the processes of transcription, translation, mRNA degradation and protein degradation, and plot the contour maps for the CVs (Fig. 7). The CVs change monotonically with increasing *D* but non-monotonically with *τ*. The CVs are of the expected values in the light red regions. Clearly, the possible *D* and *τ* should be located in the overlap of these four regions (i.e., dark red region), suggesting that a long correlation time in the CN is probably necessary for the p53 variability observed experimentally.

**Figure 8:**
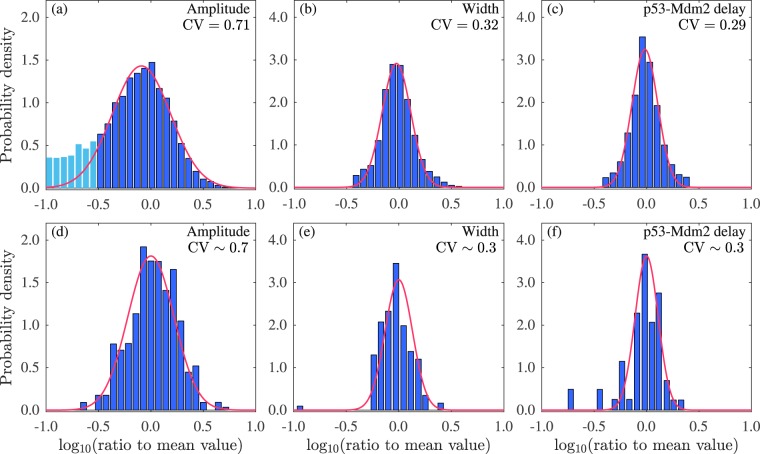
Comparison of simulation results with the experimental data. The upper row shows the histograms for the base-10 logarithm of the peak amplitude and width of p53 oscillation and p53-Mdm2 delay divided by their respective mean values, respectively. The lower row shows the corresponding experimental data from ref.^11^. 1000 runs of stochastic simulations lasting 3000 min are performed with *S* = 0.3, Ω = 10^4^, *D* = 0.04, and *τ* = 1000 min. Red curves are fittings by Gaussian density function (2*πσ*^2^)^−1/2^exp[−(*x* − *μ*)^2^/(2*σ*^2^)] with (*μ*, *σ*) = (−0.091, 0.28) (**a**), (−0.026, 0.14) (**b**), (−0.017, 0.12) (**c**), (0, 0.22) (**d**), (0, 0.13) (**e**), and (0, 0.11) (**f**). The average CVs are marked in the figure. For (**a**)–(**c**), the CV can be estimated as follows: CV = *σ*_0_/*μ*_0_, where ${\mu }_{0}=\exp [(\mu +lo{g}_{10}\bar{x})\mathrm{ln}\,10+{(\sigma \mathrm{ln}10)}^{2}/2]$, ${\sigma }_{0}^{2}=\{\exp [{(\sigma \mathrm{ln}10)}^{2}]-1\}\exp [2(\mu +lo{g}_{10}\bar{x})\mathrm{ln}\,10+{(\sigma \mathrm{ln}10)}^{2}]$, and $\bar{x}$ is the corresponding mean value: $\bar{x}=11761$ for amplitude, 118 min for width, and 101 min for p53-Mdm2 delay.

To justify our estimates of noise parameters, i.e., *S* = 0.3, Ω = 10^4^, and (*D*, *τ*) in the above overlap region, we take *D* = 0.04 and *τ* = 10^3^ min and perform 1000 runs of stochastic simulations with intrinsic noise, extrinsic noise (in transcription, translation, and degradation of mRNA and protein) and cellular heterogeneity (in *n*_DSB_ and 29 rate constants). Consequently, 54% of simulation trajectories of [p53] display sustained oscillation. The histograms in Figs. 8 and S4 can be roughly fitted by normal distributions (we also check the normality using Q-Q plot). We then calculate the average CVs (see *SI Text*): 0.71, 0.32, 0.26, and 0.29 for the amplitude, width, period and p53-Mdm2 delay, respectively. All these results agree well with the experimental data (Fig. 8(d–f), cited from ref.^11^ the CV of the period was only mentioned in the main text therein). Notably, the histogram for the amplitude is left skewed due to the difficulty in distinguishing irregular small fluctuations from sustained oscillations. To fit the normal distribution to the majority of the data, light blue bars with the values less than −0.5 are excluded from the fitting (Fig. 8(a)). Taken together, appropriate cellular heterogeneity and extrinsic colored noise with proper strength and correlation time suffice to induce the observed variability in p53 oscillations.

## Discussion and Conclusion

One striking feature of p53 oscillation is the variability in amplitude and period. Unraveling the underlying mechanism for this variability is essential to understand the modulation of p53 dynamics. Diverse sources of stochastic factors may contribute to that variability. We found that intrinsic noise, extrinsic noise and cellular heterogeneity have distinct roles. Given large amounts of network components, intrinsic noise has a minor effect on the variability. In contrast, extrinsic colored noise and cellular heterogeneity can induce a marked variability. With all these factors combined, simulation results quantitatively agree with the experimental observations^11^.

Our analyses of p53 oscillations under various stochastic conditions reveal an approximate relationship between the CVs for the combined and individual noise sources, which can facilitate estimation of noise parameters, such as the strength and correlation time of noise. Our work provides new insights into the underlying mechanism for p53 variability. In contrast to the conclusion in refs.^13,15^, where the numbers of proteins are assumed to be only 10^2^, the intrinsic noise does not contribute much to p53 fluctuations given the actual numbers of proteins and mRNAs are nearly 10^4^ and 10^3^, respectively. Cellular heterogeneity affects whether cells can exhibit p53 oscillations, but it alone fails to underlie the stochastic dynamics in individual cells. Variability in individual trajectories of p53 oscillation is mainly attributed to the extrinsic colored noise with proper strength and correlation time. Moreover, the CVs show a non-monotonic dependence on the correlation time of colored noise, which may be a generic feature of biochemical oscillators.

Similar to a theoretical study^13^, here we present a minimal model of the core regulatory network of p53 dynamics. The model comprises the ATM-p53-Wip1 and p53-Mdm2 negative feedback loops, and the synthesis of mRNAs is explicitly modeled, which increases the effective time delay in negative feedback. Therefore, p53 oscillations can arise in a relatively wide parameter range. Taking into account other processes such as DNA repair, regulation of cell cycle progression, and crosstalk between the p53 and other signaling pathways should advance our understanding of p53 oscillations and p53 variability. Such modeling deserves more efforts given p53 dynamics play a critical role in its tumor suppressive function.

## Supplementary information

Supplementary information
