# Supplementary material for: Roles of cellular heterogeneity, intrinsic and extrinsic noise in variability of p53 oscillation
Source: Sci Rep. 2019 Apr 10;9:5883. doi: 10.1038/s41598-019-41904-9 (PMC6458166; doi:10.1038/s41598-019-41904-9)
Supplement: Supplementary file 1 — Supplementary information [file 41598_2019_41904_MOESM1_ESM.pdf]

Supplementary Information

Roles of cellular heterogeneity, intrinsic and extrinsic noise in variability of p53 oscillation

Dao-Guang Wang, Shaobing Wang, Bo Huang and Feng Liu

This document includes 1) Supplementary Text describing the simulation algorithm, 2) three Supplementary Tables, and 3) four Supplementary Figures.

I. SUPPLEMENTARY TEXT

1. ORDINARY DIFFERENTIAL EQUATIONS FOR THE MODEL

Denote the volume of a cell by V, and the number of species s by $N_{\rm s}$, where $\rm s \in \mathbb{S} = \{p53_u, p53_p, Mdm2_n, Mdm2_c, Wip1, ATM, ATM_2, ATM_p, p53\ mRNA, mdm2\ mRNA, wip1\ mRNA\}$. Set $c_{\rm s} = N_{\rm s}/V$ to be the concentration of species s in units of volume^{-1} and Z to be a positive constant in units of volume^{-1} as a unit concentration (also called simulation unit in the literature). Then, $[X_{\rm s}] = c_{\rm s}/Z$ is the dimensionless concentration of species s. That is, $[X_{\rm s}] = N_{\rm s}/\Omega$, and $\Omega = ZV$ could be regarded as the dimensionless volume of a cell.

According to Refs. [1, 2], the kinetic equations for ATM activation are as follows:

$$\begin{aligned} \frac{d[\mathrm{ATM_p}]}{dt} &= k_{\rm acatm}\frac{n_{\rm DSB}}{n_{\rm DSB}+j_{\rm dsb}}\frac{[\mathrm{ATM_p}][\mathrm{ATM}]}{[\mathrm{ATM}]+j_{\rm acatm}} + k_{\rm auto}\frac{[\mathrm{ATM}]}{[\mathrm{ATM}]+j_{\rm atm}} \\ &\quad -k_{\rm deatmwip}\frac{[\mathrm{Wip1}][\mathrm{ATM_p}]}{[\mathrm{ATM_p}]+j_{\rm deatmwip}} - k_{\rm deatm}\frac{[\mathrm{ATM_p}]}{[\mathrm{ATM_p}]+j_{\rm deatm}} \qquad (1) \\ \frac{d[\mathrm{ATM_2}]}{dt} &= k_{\rm dim}[\mathrm{ATM}]^2 - k_{\rm udim}[\mathrm{ATM_2}] \qquad (2) \end{aligned}$$

ATM denotes dephosphorylated ATM monomer. Since the total amount of ATM does not change markedly in the DNA damage response [3, 4], $[\mathrm{ATM}] + [\mathrm{ATM_p}] + 2[\mathrm{ATM_2}]$ is considered a constant. The binding and disassociation reactions are described based on the law of mass action, while the enzymatic reactions are characterized by the Michaelie-Menten kinetics. $n_{\rm DSB}$ denotes the number of DSBs.

The dynamics of p53 are described as follows:

$$\begin{aligned} \frac{d[\mathrm{p53\ mRNA}]}{dt} &= k_{\rm sp53} - k_{\rm dp53m}[\mathrm{p53\ mRNA}] \qquad (3) \\ \frac{d[\mathrm{p53_u}]}{dt} &= k_{\rm rp53}[\mathrm{p53\ mRNA}] - k_{\rm bdp53}[\mathrm{p53_u}] - k_{\rm acp531}\frac{[\mathrm{ATM_p}][\mathrm{p53_u}]}{[\mathrm{p53_u}]+j_{\rm ap53}} \\ &\quad +k_{\rm wip53s}\frac{[\mathrm{Wip1}][\mathrm{p53_p}]}{[\mathrm{p53_p}]+j_{\rm wip53s}} - k_{\rm dp53}\frac{[\mathrm{Mdm2_n}][\mathrm{p53_u}]}{[\mathrm{p53_u}]+j_{\rm p53}} \qquad (4) \\ \frac{d[\mathrm{p53_p}]}{dt} &= k_{\rm acp531}\frac{[\mathrm{ATM_p}][\mathrm{p53_u}]}{[\mathrm{p53_u}]+j_{\rm ap53}} - k_{\rm bdp53p}[\mathrm{p53_p}] - k_{\rm dp53s}\frac{[\mathrm{Mdm2_n}][\mathrm{p53_p}]}{[\mathrm{p53_p}]+j_{\rm p53s}} - k_{\rm wip53s}\frac{[\mathrm{Wip1}][\mathrm{p53_p}]}{[\mathrm{p53_p}]+j_{\rm wip53s}} \qquad (5) \end{aligned}$$

where $\mathrm{p53_u}$ and $\mathrm{p53_p}$ denote nuclear unphosphorylated (inactive) p53 and phosphorylated (active) p53, respectively. These equations describe the basal transcription, translation, degradation, and reversible phosphorylation of p53. As in Refs. [1, 2], the transcription rate of p53 is considered a constant since p53 levels are primarily modulated by posttranslational modifications.

For Mdm2,

$$\frac{d[mdm2\ \text{mRNA}]}{dt} = k_{\text{smdm20}} - k_{\text{dmdm2m}}[mdm2\ \text{mRNA}] + k_{\text{smdm2}}\frac{[\text{p53}_\text{p}]^4}{[\text{p53}_\text{p}]^4 + j_{\text{smdm2}}^4} \tag{6}$$

$$\frac{d[\text{Mdm2}_\text{c}]}{dt} = k_{\text{rmdm2}}[mdm2\ \text{mRNA}] + k_\text{o}[\text{Mdm2}_\text{n}] - k_\text{i}[\text{Mdm2}_\text{c}] - k_{\text{dmdm2c}}[\text{Mdm2}_\text{c}] \tag{7}$$

$$\frac{d[\text{Mdm2}_\text{n}]}{dt} = k_\text{i}[\text{Mdm2}_\text{c}] - k_\text{o}[\text{Mdm2}_\text{n}] - k_{\text{dmdm2n1}}\frac{[\text{ATM}_\text{p}][\text{Mdm2}_\text{n}]}{[\text{Mdm2}_\text{n}] + j_{\text{mdm2n}}} - k_{\text{dmdm2n0}}[\text{Mdm2}_\text{n}] \tag{8}$$

$$\tag{9}$$

Both cytoplasmic Mdm2 (Mdm2_c) and nuclear Mdm2 (Mdm2_n) are considered here. The transcription of *mdm2* by p53_p is described by Hill function, while the shuttling of Mdm2 between the nucleus and cytoplasm is described by linear terms.

Similarly, the dynamics of Wip1 are governed by

$$\frac{d[wip1\ \text{mRNA}]}{dt} = k_{\text{swip10}} - k_{\text{dwip1m}}[wip1\ \text{mRNA}] + k_{\text{swip1}}\frac{[\text{p53}_\text{p}]^3}{[\text{p53}_\text{p}]^3 + j_{\text{swip1}}^3} \tag{10}$$

$$\frac{d[\text{Wip1}]}{dt} = k_{\text{rwip1}}[wip1\ \text{mRNA}] - k_{\text{dwip1}}[\text{Wip1}] \tag{11}$$

All the parameter values and initial values of variables are listed in Table S1 and S2, respectively.

2. BINOMIAL τ-LEAP ALGORITHM

Consider a well-stirred chemical reaction system which contains N molecular species with the number $X_i(t)$ of species S_i $(i = 1, 2, ..., N)$ at time t. They participate in M chemical reactions denoted by R_j $(j = 1, 2, ..., M)$. For each reaction R_j, v_{ij} represents the change in the number of species S_i due to the occurrence of reaction R_j, and $a_j\mathbf{X}(t))$, with $\mathbf{X}(t) = (X_1(t), X_2(t), ..., X_N(t))$, is the propensity function, i.e., $a_j(\mathbf{X}(t))dt$ is the probability that reaction R_j occurs in the infinitesimal time interval $[t, t + dt)$.

Some reactions can lead to a reduction in the number of species, such as degradation or transition of species between distinct forms. Denote these reactions by R_j $(j = 1, 2, ..., m)$, and there is at least one $v_{ij} < 0$ $(i = 1, 2, ..., N)$ for each of them. Denote the other reactions by R_j $(j = m + 1, m + 2, ..., M)$. At time t, the number of occurrence, k_j, for reactions R_j $(j = m + 1, m + 2, ..., M)$ in the following time interval τ obeys the Poisson distribution

$$p_\text{P}(k_j) = \frac{e^{-a_j(\mathbf{X}(t))\tau}}{k_j!}[a_j(\mathbf{X}(t))\tau]^{k_j}, j = m + 1, m + 2, ..., M, \tag{12}$$

while that for reactions R_j $(j = 1, 2, ..., m)$ satisfies the binomial distribution

$$p_\text{B}(k_j) = \frac{k_{j,\max}!}{k_j!(k_{j,\max} - k_j)!}p^{k_j}(1 - p)^{k_{j,\max} - k_j}, j = 1, 2, ..., m, \tag{13}$$

where $k_{j,\max}$ is the maximal number of occurrence of reaction R_j, which is determined by the number of the species that can be consumed completely due to reaction R_j, and $p = a_j(\mathbf{X}(t))\tau/k_{j,\max}$ is the probability that reaction R_j occurs within $[t, t + \tau)$. Notably, if $k_{j,\max}$ is sufficiently large and p is small enough, the binomial distribution can be approximated by the Poisson distribution.

The stochastic simulation algorithm in our work is as follows:

(1) Initialize the time $t = 0$ and system state $\mathbf{X} = \mathbf{X}(0)$.

(2) Repeat steps 3–6 until the simulation is finished.

(3) Compute $a_j(\mathbf{X}(t))$.

(4) For $j = 1, 2, ...m$, find $k_{j,\max}$ and sample k_j from the binomial distribution (Eq (13)) in a sequence, e.g., from $j = 1$ to m. After k_j is sampled, k_{j+1} is sampled with $\mathbf{X}^{(j)}(t) := \mathbf{X}^{(j)}(t) + \sum_{i=1}^{N} v_{ij}k_j$ replacing $\mathbf{X}(t)$ in p, where $\mathbf{X}^{(0)}(t) = \mathbf{X}(t)$.

(5) For $j = m + 1, m + 2, ..., M$, sample k_j from the Poisson distribution (Eq. (12)).

(6) Update time to $t + \tau$ and state by $X_i(t + \tau) = X_i(t) + v_{ij}k_j$, for $i = 1, 2, ..., N$, with $\tau = 0.01$ min.

3. METHOD FOR CALCULATING COEFFICIENT OF VARIANCE IN FIG. 8

If a random variable, X, obeys a log-normal distribution, then the logarithmics of X, $Y = \ln X$, should follow a normal distribution. For a set of data x, if the histogram of $y = \ln x$ can be fit with the normal distribution with mean μ and standard deviation σ, then the mean and variance of x should be $\exp(\mu + \sigma^2/2)$ and $[\exp(\sigma^2) - 1]\exp(2\mu + \sigma^2)$, respectively. Similarly, if the histogram of $y = \log_{10}(x/\bar{x})$ can be fit with the normal distribution with mean μ and standard deviation σ, then we can rewrite $y = \log_{10}(x/\bar{x})$ as $(y + \log_{10}\bar{x})\ln 10 = \ln x$. Denote $z = (y + \log_{10}\bar{x})\ln 10 = \ln x$, then z can be fit with the normal distribution with mean $(\mu + \log_{10}\bar{x})\ln 10$ and standard deviation $\sigma \ln 10$. Thus, the mean and variance of x are $\exp[(\mu + \log_{10}\bar{x})\ln 10 + (\sigma \ln 10)^2/2]$ and $\{\exp[(\sigma \ln 10)^2] - 1\}\exp[2(\mu + \log_{10}\bar{x})\ln 10 + (\sigma \ln 10)^2]$, respectively.

II. SUPPLEMENTARY TABLES

TABLE S1: The default values of parameters

Index	Parameter	Description	Value	Reference
0	n_{DSB}	Number of DNA double-strand breaks (DSBs)	175	[1, 2, 6]
1	k_{dwip1}	Degradation rate constant of Wip1	0.096	[2, 8, 9]
2	k_{rwip1}	Translation rate constant of *wip1* mRNA	0.48	[7, 8]
3	k_{dwip1m}	Degradation rate constant of *wip1* mRNA	0.048	[7, 8]
4	k_{swip1}	p53-dependent transcription rate constant of *wip1* gene	0.008	[2, 8, 9]
5	k_{swip10}	Basal transcription rate constant of *wip1* gene	0.0008	[2, 8, 9]
6	k_{dmdm2n1}	ATM-mediated auto-degradation rate constant of nuclear Mdm2	0.096	[1, 2, 6]
7	k_{dmdm2n0}	Basel degradation rate constant of nuclear Mdm2	0.0032	[1, 2, 6]
8	k_{i}	Nuclear import rate constant of Mdm2	0.08	[1, 2, 6]
9	k_{o}	Nuclear export rate constant of Mdm2	0.04	[1, 2, 6]
10	k_{dmdm2c}	Degradation rate constant of cytoplasmic Mdm2	0.0064	[1, 2, 6, 7]
11	k_{rmdm2}	Translation rate constant of *mdm2* mRNA	0.16	[7, 8]
12	k_{dmdm2m}	Degradation rate constant of *mdm2* mRNA	0.048	[7, 8]
13	k_{smdm2}	p53-dependent transcription rate constant of *mdm2* gene	0.016	[1, 2, 6, 7]
14	k_{smdm20}	Basal transcription rate constant of *mdm2* gene	0.0016	[1, 2, 6, 7]
15	k_{dp53s}	Mdm2-dependent degradation rate constant of phosphorylated p53	0.012	[1, 2, 6, 8]
16	k_{bdp53p}	Basal degradation rate constant of phosphorylated p53	0.0008	Estimated
17	k_{dp53}	Mdm2-dependent degradation rate of unphosphorelated p53	0.24	[1, 2, 6, 8]
18	k_{bdp53}	Basal degradation rate constant of unphosphorylated p53	0.04	Estimated
19	k_{acp531}	ATM-mediated phosphorylation rate constant of p53	0.08	[1, 2, 6, 7]
20	k_{wip53s}	Wip1-mediated dephosphorylation rate constant of p53	0.004	Estimated
21	k_{rp53}	Translation rate constant of *p53* mRNA	0.32	[7, 8]
22	k_{dp53m}	Degradation rate constant of *p53* mRNA	0.08	[7, 8]
23	k_{sp53}	Basal transcription rate constant of *p53* gene	0.008	[1, 2, 6, 8]
24	k_{udim}	Dissociation rate constant of $\mathrm{ATM_2}$	0.016	[1, 2, 6]
25	k_{dim}	Dimerization rate constant of ATM	0.008	[1, 6, 7]
26	k_{deatmwip}	Dephosphorylation rate constant of $\mathrm{ATM_p}$ by Wip1	0.24	Estimated
27	k_{deatm}	Wip1-independent dephosphorylation rate constant of $\mathrm{ATM_p}$	0.0008	[1, 2, 6, 8]
28	k_{auto}	Basal phosphorylation rate constant of ATM	0.016	[7, 8]
29	k_{acatm}	DSB-induced auto-phosphorylation rate constant of ATM	0.136	[1, 2, 6, 8]
30	j_{swip1}	Michaelis constant for p53-dependent transcription of *wip1* gene	0.7	[2, 8, 9]
31	j_{mdm2n}	Michaelis constant for ATM-induced auto-degradation of nuclear Mdm2	1	[1, 2, 6]
32	j_{smdm2}	Michaelis constant for p53-dependent transcription of *mdm2* gene	1	[1, 2, 6, 7]
33	j_{p53s}	Michaelis constant for Mdm2-mediated degradation of phosphorylated p53	0.02	[1, 2, 6, 8]
34	j_{p53}	Michaelis constant for Mdm2-mediated degradation of unphosphorylated p53	0.1	[1, 2, 6, 8]
35	j_{wip53s}	Michaelis constant for dephosphorylation of p53 by Wip1	0.1	Estimated
36	j_{ap53}	Michaelis constant for phosphorylation of p53 by ATM	3	[1, 2, 6, 8]
37	j_{atm}	Michaelis constant for basal phosphorylation of ATM	0.1	[1, 2, 6, 8]
38	j_{deatmwip}	Michaelis constant for dephosphorylation of ATM by Wip1	0.2	Estimated
39	j_{deatm}	Michaelis constant for Wip1-independent dephosphorylation of ATM	0.2	[1, 2, 6, 8]
40	j_{acatm}	Michaelis constant for DSB-induced auto-phosphorylation of ATM	0.1	[1, 2, 6, 8]
41	j_{dsb}	Threshold for n_{DSB} to induce auto-phosphorylation of ATM	150	Estimated

Note: The 1st–29th parameters are in units of min^{-1}, while the others are dimensionless.

TABLE S2: The initial values of variables

Variable	Value	Variable	Value
[p53 mRNA]	0.1	[$\mathrm{p53_u}$]	0.046
[$\mathrm{p53_p}$]	0.001	[mdm2 mRNA]	0.033
[$\mathrm{Mdm2_c}$]	0.194	[$\mathrm{Mdm2_n}$]	0.286
[wip1 mRNA]	0.017	[Wip1]	0.083
[$\mathrm{ATM_2}$]	0.3	[$\mathrm{ATM_p}$]	0.147
[ATM]	0.253		

TABLE S3: Reaction channels and the corresponding reaction propensities

Index	Reaction	Propensity (a_j)
1	$\mathrm{Wip1} \rightarrow \Phi$	$a_1{=}k_{\mathrm{dwip1}}X_{\mathrm{Wip1}}$
2	$* \xrightarrow{\textit{wip1}\ \mathrm{mRNA}} \mathrm{Wip1}$	$a_2{=}k_{\mathrm{rwip1}}X_{\mathrm{wip1\ mRNA}}$
3	$\textit{wip1}\ \mathrm{mRNA} \rightarrow \Phi$	$a_3{=}k_{\mathrm{dwip1m}}X_{\mathrm{wip1\ mRNA}}$
4	$* \xrightarrow{\mathrm{p53_p}} \textit{wip1}\ \mathrm{mRNA}$	$a_4{=}k_{\mathrm{swip1}}\Omega\frac{X^3_{\mathrm{p53_p}}}{X^3_{\mathrm{p53_p}}+(j_{\mathrm{swip1}}\Omega)^3}$
5	$* \rightarrow \textit{wip1}\ \mathrm{mRNA}$	$a_5{=}k_{\mathrm{swip10}}\Omega$
6	$\mathrm{Mdm2_n} \xrightarrow{\mathrm{ATM_p}} \Phi$	$a_6{=}k_{\mathrm{dmdm2n1}}\frac{X_{\mathrm{ATM_p}}X_{\mathrm{Mdm2_n}}}{X_{\mathrm{Mdm2_n}}+j_{\mathrm{mdm2n}}\Omega}$
7	$\mathrm{Mdm2_n} \rightarrow \Phi$	$a_7{=}k_{\mathrm{dmdm2n0}}X_{\mathrm{Mdm2_n}}$
8	$\mathrm{Mdm2_c} \rightarrow \mathrm{Mdm2_n}$	$a_8{=}k_{\mathrm{i}}X_{\mathrm{Mdm2_c}}$
9	$\mathrm{Mdm2_n} \rightarrow \mathrm{Mdm2_c}$	$a_9{=}k_{\mathrm{o}}X_{\mathrm{Mdm2_n}}$
10	$\mathrm{Mdm2_c} \rightarrow \Phi$	$a_{10}{=}k_{\mathrm{dmdm2c}}X_{\mathrm{Mdm2_c}}$
11	$* \xrightarrow{\textit{mdm2}\ \mathrm{mRNA}} \mathrm{Mdm2_c}$	$a_{11}{=}k_{\mathrm{rmdm2}}X_{\mathrm{mdm2\ mRNA}}$
12	$\textit{mdm2}\ \mathrm{mRNA} \rightarrow \Phi$	$a_{12}{=}k_{\mathrm{dmdm2m}}X_{\mathrm{mdm2\ mRNA}}$
13	$* \xrightarrow{\mathrm{p53_p}} \textit{mdm2}\ \mathrm{mRNA}$	$a_{13}{=}k_{\mathrm{smdm2}}\Omega\frac{X^4_{\mathrm{p53_p}}}{X^4_{\mathrm{p53_p}}+(j_{\mathrm{smdm2}}\Omega)^4}$
14	$* \rightarrow \textit{mdm2}\ \mathrm{mRNA}$	$a_{14}{=}k_{\mathrm{smdm20}}\Omega$
15	$\mathrm{p53_p} \xrightarrow{\mathrm{Mdm2_n}} \Phi$	$a_{15}{=}k_{\mathrm{dp53s}}\frac{X_{\mathrm{Mdm2_n}}X_{\mathrm{p53_p}}}{X_{\mathrm{p53_p}}+j_{\mathrm{p53s}}\Omega}$
16	$\mathrm{p53_p} \rightarrow \Phi$	$a_{16}{=}k_{\mathrm{bdp53p}}X_{\mathrm{p53_p}}$
17	$\mathrm{p53_u} \xrightarrow{\mathrm{Mdm2_n}} \Phi$	$a_{17}{=}k_{\mathrm{dp53}}\frac{X_{\mathrm{Mdm2_n}}X_{\mathrm{p53_u}}}{X_{\mathrm{p53_u}}+j_{\mathrm{p53_u}}\Omega}$
18	$\mathrm{p53_u} \rightarrow \Phi$	$a_{18}{=}k_{\mathrm{dwip1m}}X_{\mathrm{p53_u}}$
19	$\mathrm{p53_u} \xrightarrow{\mathrm{ATM_p}} \mathrm{p53_p}$	$a_{19}{=}k_{\mathrm{acp531}}\frac{X_{\mathrm{ATM_p}}X_{\mathrm{p53_u}}}{X_{\mathrm{p53_u}}+j_{\mathrm{ap53}}\Omega}$
20	$\mathrm{p53_p} \xrightarrow{\mathrm{Wip1}} \mathrm{p53_u}$	$a_{20}{=}k_{\mathrm{wip53s}}\frac{X_{\mathrm{Wip1}}X_{\mathrm{p53_p}}}{X_{\mathrm{p53_p}}+j_{\mathrm{wip53s}}\Omega}$
21	$* \xrightarrow{\textit{p53}\ \mathrm{mRNA}} \mathrm{p53_u}$	$a_{21}{=}k_{\mathrm{rp53}}X_{\mathrm{p53\ mRNA}}$
22	$\textit{p53}\ \mathrm{mRNA} \rightarrow \Phi$	$a_{22}{=}k_{\mathrm{dp53m}}X_{\mathrm{p53\ mRNA}}$
23	$* \rightarrow \textit{p53}\ \mathrm{mRNA}$	$a_{23}{=}k_{\mathrm{sp53}}\Omega$
24	$\mathrm{ATM_2} \rightarrow 2\mathrm{ATM}$	$a_{24}{=}k_{\mathrm{udim}}X_{\mathrm{ATM_2}}$
25	$2\mathrm{ATM} \rightarrow \mathrm{ATM_2}$	$a_{25}{=}k_{\mathrm{dim}}X^2_{\mathrm{ATM}}/\Omega$
26	$\mathrm{ATM_p} \xrightarrow{\mathrm{Wip1}} \mathrm{ATM}$	$a_{26}{=}k_{\mathrm{deatmwip}}\frac{X_{\mathrm{Wip1}}X_{\mathrm{ATM_p}}}{X_{\mathrm{ATM_p}}+j_{\mathrm{deatmwip}}\Omega}$
27	$\mathrm{ATM_p} \rightarrow \mathrm{ATM}$	$a_{27}{=}k_{\mathrm{deatm}}\Omega\frac{X_{\mathrm{ATM_p}}}{X_{\mathrm{ATM_p}}+j_{\mathrm{deatm}}\Omega}$
28	$\mathrm{ATM} \rightarrow \mathrm{ATM_p}$	$a_{28}{=}k_{\mathrm{auto}}\Omega\frac{X_{\mathrm{ATM}}}{X_{\mathrm{ATM}}+j_{\mathrm{atm}}\Omega}$
29	$\mathrm{ATM} \xrightarrow{n_{\mathrm{DSB}},\mathrm{ATM_p}} \mathrm{ATM_p}$	$a_{29}{=}k_{\mathrm{acatm}}\frac{n_{\mathrm{DSB}}}{n_{\mathrm{DSB}}+j_{\mathrm{dsb}}}\frac{X_{\mathrm{ATM_p}}X_{\mathrm{ATM}}}{X_{\mathrm{ATM}}+j_{\mathrm{acatm}}\Omega}$

Note: $*$ and Φ denote the production and degradation,respectively.

TABLE S4: Parameters perturbed in the simulation of extrinsic noise

Process	Species	Parameter	Noise
Transcription	p53	k_{sp53}	$\eta_1(t)$
	Mdm2	k_{smdm20}, k_{smdm2}	$\eta_2(t)$
	Wip1	k_{swip10}, k_{swip1}	$\eta_3(t)$
Translation	p53	k_{rp53}	$\eta_4(t)$
	Mdm2	k_{rmdm2}	$\eta_5(t)$
	Wip1	k_{rwip1}	$\eta_6(t)$
mRNA degradation	p53	k_{dp53m}	$\eta_7(t)$
	Mdm2	k_{dmdm2m}	$\eta_8(t)$
	Wip1	k_{dwip1m}	$\eta_9(t)$
Protein degradation	p53	k_{dwip1m}, k_{dp53}, k_{bdp53p}, k_{dp53s}	$\eta_{10}(t)$
	Mdm2	k_{dmdm2c}, k_{dmdm2n0}, k_{dmdm2n1}	$\eta_{11}(t)$
	Wip1	k_{dwip1}	$\eta_{12}(t)$

[1] Zhang, X.-P., Liu, F., Cheng, Z. & Wang, W. Cell fate decision mediated by p53 pulses. *Proc. Natl. Acad. Sci. USA* **106**, 12245-12250 (2009).
[2] Zhang, X.-P., Liu, F. & Wang, W. Two-phase dynamics of p53 in the DNA damage response. *Proc. Natl. Acad. Sci. USA* **108**, 8990-8995 (2011).
[3] Banin, S. *et al.* Enhanced phosphorylation of p53 by ATM in response to DNA damage. *Science* **281**, 1674-1677 (1998).
[4] Canman, C. E. *et al.* Activation of the ATM kinase by ionizing radiation and phosphorylation of p53. *Science* **281**, 1677-1679 (1998).
[5] Feng, X.-J. *et al.* Optimizing Genetic Circuits by Global Sensitivity Analysis. *Biophys. J.* **87**, 2195-2022 (2004).
[6] Ma, L. *et al.* A plausible model for the digital response of p53 to DNA damage. *Proc. Natl. Acad. Sci. USA* **102**, 14266-14271 (2005).
[7] Sun, T., Yang, W., Liu, J. & Shen, P. P. Modeling the basal dynamics of p53 system. *PloS One* **6**, e27882 (2011).
[8] Eliaš, J., Dimitrio, L., Clairambault, J. & Natalini, R. The p53 protein and its molecular network: modelling a missing link between DNA damage and cell fate. *Biochim. Biophys. Acta, Proteins Proteomics* **1844**, 232-247 (2014).
[9] Batchelor, E. *et al.* Recurrent initiation: a mechanism for triggering p53 pulses in response to DNA damage. *Mol. Cell* **30**, 277-289 (2008).

III. SUPPLEMENTARY FIGURES

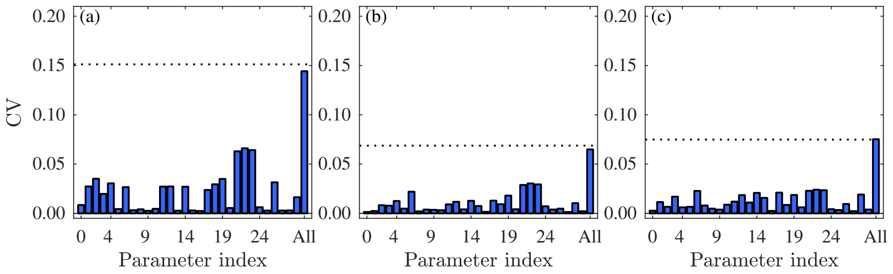

FIG. S1: Influence of cellular heterogeneity on p53 oscillation. The CV for the peak width (**a**) and period (**b**) of p53 pulses as well as p53-Mdm2 delay (**c**) when one parameter alone (0–29) or all parameters are perturbed simultaneously (All). The dotted line denotes the estimate of the CV in the latter case. Parameters are indexed as follows: 0. n_{DSB}, 1. k_{dwip1}, 2. k_{rwip1}, 3. k_{dwip1m}, 4. k_{swip1}, 5. k_{swip10}, 6. k_{dmdm2n1}, 7. k_{dmdm2n0}, 8. k_{i}, 9. k_{o}, 10. k_{dmdm2c}, 11. k_{rmdm2}, 12. k_{dmdm2m}, 13. k_{smdm2}, 14. k_{smdm20}, 15. k_{dp53s}, 16. k_{bdp53p}, 17. k_{dp53}, 18. k_{bdp53}, 19. k_{acp531}, 20. k_{wip53s}, 21. k_{rp53}, 22. k_{dp53m}, 23. k_{sp53}, 24. k_{udim}, 25. k_{dim}, 26. k_{deatmwip}, 27. k_{deatm}, 28. k_{auto}, and 29. k_{acatm}.

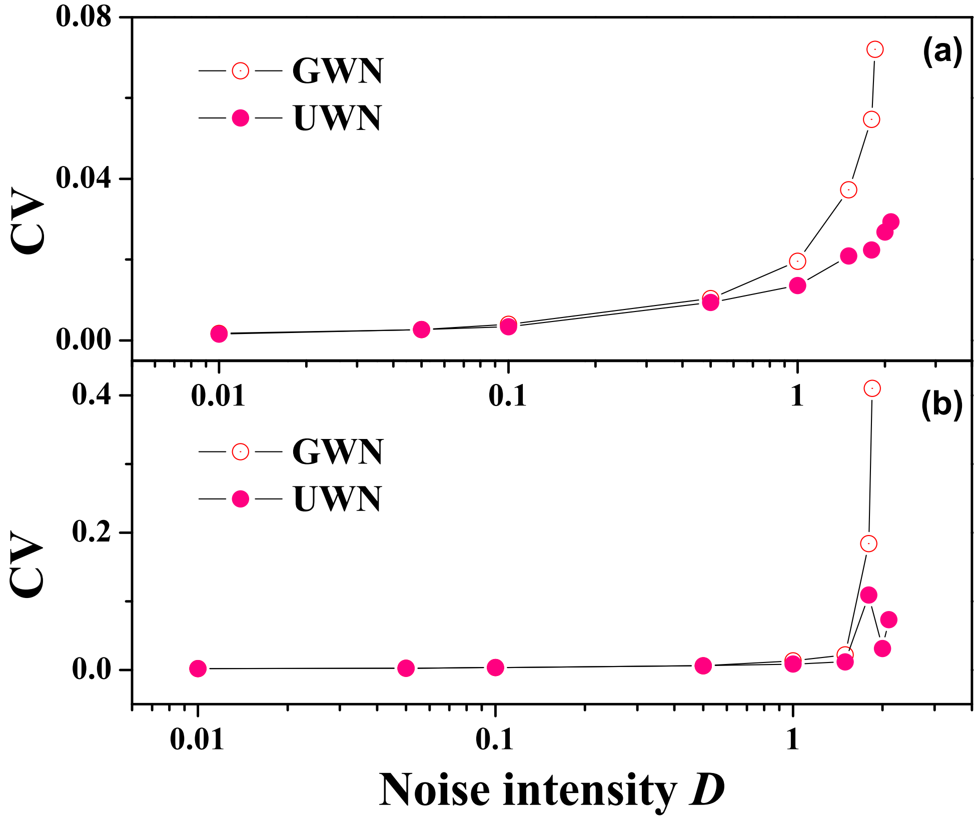

FIG. S2: Dependence of the CVs on the intensity of extrinsic noise. Only Gaussian white noise (GWN) or uniform white noise (UWN) is applied to the transcription of *p53*, *mdm2* and *wip1*. Comparison of the CVs of the amplitude (**a**) and period (**b**) of p53 pulses. In each case, 1000 runs of stochastic simulations lasting 3000 min are performed. The axis of D is on logarithmic scale.

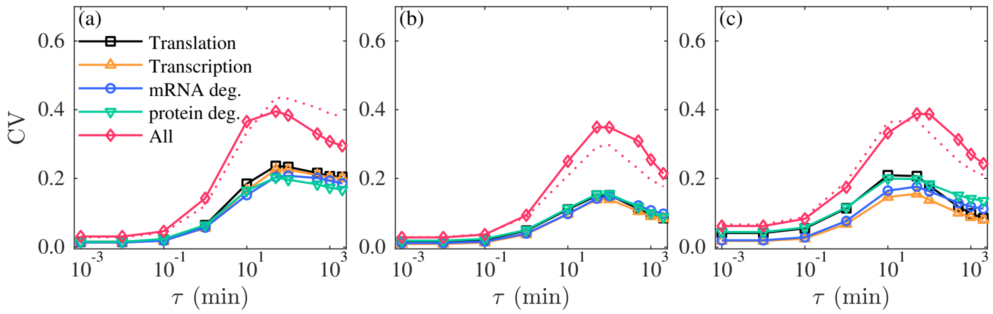

FIG. S3: Influence of extrinsic colored noise on variability in oscillations. Comparison of the CV for width (**a**) and period (**b**) of p53 pulses as well as p53-Mdm2 delay (**c**) when the CN is included in transcription, translation, mRNA degradation, protein degradation alone or together at $D = 0.1$. The dotted line denotes the estimate for the CV in the latter case. In each case, 1000 runs of stochastic simulations lasting 3000 min are performed. The axis of τ is on logarithmic scale.

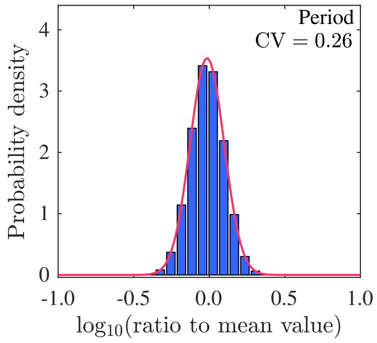

FIG. S4: Histogram for the base-10 logarithm of the peak period of p53 oscillation divided by the mean value. 1000 runs of stochastic simulations lasting 3000 min are performed at $S = 0.3$, $\Omega = 10^4$, $D = 0.04$, and $\tau = 1000$ min. Red curves are fittings by Gaussian density function $(2\pi\sigma^2)^{-1/2}\exp[-(x-\mu)^2/(2\sigma^2)]$ with $\mu = -0.015$ and $\sigma = 0.11$. The CV can be estimated at μ and σ: CV $= \sigma_0/\mu_0$, with $\mu_0 = \exp[(\mu+\log_{10}\bar{x})\ln 10+(\sigma\ln 10)^2/2]$, $\sigma_0^2 = \{\exp[(\sigma\ln 10)^2]-1\}\exp[2(\mu+\log_{10}\bar{x})\ln 10+(\sigma\ln 10)^2]$, and $\bar{x} = 312$ min is the corresponding mean value.
